# 간호사의 내분비계 교란 물질 노출, 감정 노동, 동료 지지가 월경 전 증후군에 미치는 영향

**DOI:** 10.4069/kjwhn.2020.06.18

**Published:** 2020-06-23

**Authors:** Hye Young Chang, SoMi Park

**Affiliations:** 1 Division of Nursing, Wonju Severance Christian Hospital, Wonju, Korea; 1 원주세브란스기독병원 간호국; 2 Department of Nursing, Yonsei University Wonju College of Medicine, Wonju, Korea; 2 연세대학교 원주의과대학 간호학과

**Keywords:** Endocrine disruptors, Nurses, Premenstrual syndrome, Psychological burnout, Social support, 내분비계 교란 물질, 간호사, 월경 전 증후군, 감정 노동, 동료 지지

## Abstract

**Purpose:**

This study aimed to investigate the effects of exposure to endocrine disruptors, burnout, and social support from peers on premenstrual syndrome (PMS) in nurses.

**Methods:**

This descriptive correlational study was conducted among 122 nurses under the age of 49 working at a university hospital. The participants answered self-report questionnaires. The data were analyzed using the t-test, analysis of variance, Pearson or Spearman correlation coefficients, and hierarchical multiple regression in SPSS version 23.0.

**Results:**

The mean age of the nurses was 28.9 years. Of these nurses, 49.2% were working in a general ward, 24.6% in the intensive care unit, 14.8% in the emergency room, and 11.4% in an outpatient department. The explanatory power of the model was 38.3%, and it was statistically significant (F=11.74, p≤.001). Exposure to endocrine disruptors (β=0.32, *p*<.001) was the most powerful variable affecting PMS, followed by burnout (β=0.27, *p*=.001), working in the intensive care unit or emergency room (β=0.22, *p*=.003), family history of PMS (β=0.19, *p*=.009), and support from coworkers (β=–0.15, *p*=.043).

**Conclusion:**

Based on these findings, it is necessary to develop an intervention program to reduce the symptoms of PMS. Additionally, further studies are needed to develop and evaluate measures to minimize exposure to endocrine disruptors and burnout in order to alleviate PMS among nurses.

## Introduction

### 연구 필요성

월경은 여성의 생식능력을 상징하는 특성 중 하나로 초경에서부터 폐경까지 주기적으로 반복해서 나타나는 현상이다. 그러나 여성들은 월경 전 약 2–10일 사이에 신체적으로는 가스 팽만, 유방 팽만과 통증, 골반통, 체중 증가, 배변 장애, 피로, 수면 장애를, 정서적으로는 짜증, 화, 우울감, 불안, 감정 조절의 어려움을, 또한 이외에도 흥미 저하, 대인 기피, 일탈적이고 공격적인 행동 등의 월경 전 증후군을 겪을 수 있다[1]. 월경 전 증후군은 가임기 여성의 80%가 경험하며[2], 이 중 20% 이상은 학교나 직장의 출근, 인간관계 등의 일상생활에 장애를 초래할 정도로 심한 월경 전 증후군의 증상을 경험하며 삶의 질에도 영향을 미치고 있다[3].

월경 전 증후군 관련 요인에 관한 선행 연구 결과를 살펴보면 부정적인 월경 태도, 다량의 월경량, 정상보다 긴 월경 주기 및 월경통[4]과 같은 월경 특성이 월경 전 증후군과 관련 있는 것으로 보고하고 있다. 최근에는 인체 내로 들어와 마치 호르몬과 같이 작용하면서 정상 호르몬의 기능을 방해하는 물질로 알려진 내분비계 교란 물질이 월경 주기 변화, 조기 초경, 월경 곤란증, 월경 전 증후군 등 여성의 생식 건강 관련 질환을 증가시킨다고 알려졌다[5]. 이러한 내분비계 교란 물질은 우리가 일상생활에서 흔히 사용하는 플라스틱 제품, 세제, 비닐봉지 등에서 배출되며, 이러한 제품 사용 횟수의 증가가 월경 전 증후군의 발생과 관계가 있는 것으로 보고되고 있다[6]. 월경 전 증후군의 원인은 불확실하지만 혈장 내 에스트로겐과 프로게스테론 등의 스테로이드 호르몬 수준에 대한 여성의 민감성과 내분비계의 상호작용이 중요하게 고려되고 있는데, 내분비계 교란 물질은 스테로이드 호르몬으로 모방하거나 스테로이드 호르몬의 신호에 대해 길항작용을 하여 질병 발생에 관여하는 것으로 알려져 있다[7]. 또한 내분비계나 생식기의 원인 외에도 정신적 스트레스와 같은 요인이 시상하부-뇌하수체-난소에 영향을 미치며[8], 높은 스트레스와 긴장감 및 압박감은 월경 전 증후군 증상을 더욱 악화시키게 된다[4]. 200명의 여대생을 대상으로 조사한 한 연구에서도 월경 전 증후군과 스스로 인지하는 스트레스 사이에는 매우 강한 관련성이 있다고 보고하고 있다[9]. 특히 간호사는 고객 권리 의식의 향상에 따라 정신적 긴장이 더욱 가중되고, 긴급한 상황에서는 심한 스트레스를 경험한다[10]. 뿐만 아니라 의사, 동료 간호사 및 타 부서 직원들과 상호작용하는 과정에서 자신의 감정을 표현하기 보다는 감추는 노력을 해야 하는 경우가 많아 다른 서비스 직업군과 비교하였을 때 높은 수준의 감정 노동을 경험하는 것으로 보고되고 있다[11]. 이러한 감정 노동과 스트레스는 월경 전 증후군과 유의한 정적 상관관계에 있는 것으로 보인다[12]. 이처럼 높은 수준의 감정 노동을 경험하는 간호사 집단은 상호 협동과 의존성이 두드러지는 조직의 특성상 병원의 상황을 모르는 가족이나 친구보다 동료의 지지가 매우 중요하다[13]. 동료 지지는 조직 내에서 직장 상사, 동료, 후배로부터 받을 수 있는 정서적, 물질적인 지지로, 이는 근무로 인해 발생하는 감정 노동이나 분노를 감소시키고 감정 노동의 완충 역할을 하며 스트레스를 감소시켜 정서적, 심리적 안정을 준다[14].

월경 전 증후군은 여성의 개인적인 삶뿐만 아니라 사회 경제 활동, 직장 생활에서도 장애 요인이 되어, 월경 전 증후군을 겪는 여성들은 일의 효율성과 자신감이 저하되고 정서적 소진과 자아 존중감 저하 등도 두드러지게 나타나는 것으로 보고되고 있다[15]. 그러므로 의료기관의 주요 인력인 간호사들의 월경 전 증후군을 적절히 관리하는 것은 개인의 건강 증진과 삶의 질 향상 뿐 아니라 간호업무 효율의 향상과 함께 환자들에게 질 높은 간호를 보장하는 방법이기도 하다. 이에 본 연구는 보건 의료인력의 대표적 직업군으로 가장 많은 수를 차지하는 간호사를 대상으로 월경 전 증후군 관련 요인으로 제시되는 월경 관련 특성[4], 내분비계 교란 물질 노출[6], 간호사의 직업 특성으로 인한 스트레스 요인으로 제시된 감정 노동[11] 및 이들의 완충작용을 할 수 있는 동료 지지[14]를 포함해 그 관련성을 규명함으로써 월경 전 증후군의 증상을 감소시킬 수 있는 교육 및 중재의 근거를 제시하고자 한다.

### 연구 목적

본 연구는 간호사의 내분비계 교란 물질 노출 정도, 감정 노동, 동료 지지가 월경 전 증후군에 미치는 영향을 확인함으로써 월경 전 증후군을 완화시키기 위한 중재 개발의 기초 자료를 제공하고자 하며, 이를 위한 구체적인 연구 목적은 다음과 같다.

1) 간호사의 일반적 특성, 월경 관련 특성, 내분비계 교란 물질 노출, 감정 노동, 동료 지지 및 월경 전 증후군 정도를 파악한다.

2) 일반적 특성과 월경 관련 특성에 따른 월경 전 증후군 정도의 차이를 확인한다.

3) 간호사의 월경 전 증후군과 관련 요인의 상관관계를 확인한다.

4) 간호사의 월경 전 증후군에 영향을 미치는 요인을 확인한다.

## Methods

Ethics statement: This study was approved by the Institutional Review Board of Yonsei University Wonju Severance Christian Hospital (IRB-CR318140). Informed consent was obtained from the subjects.

### 연구 설계 및 연구 대상

본 연구는 간호사의 월경 전 증후군의 영향 요인을 규명하는 상관성 조사 연구 설계이다. 본 연구의 대상자는강원도 W시 소재의 Y대학 병원에 재직하고 있는 간호사로 대상자의 수는 G power 3.1.9.2 프로그램을 이용하여 산출하였으며, 다중 회귀분석에서 유의수준 .05, 선행 연구에서 사용된 중간 효과의 크기 .15 [16], 검정력(1–β)을 80%, 독립변수를 9개로 하였을 때 118명이 산출되었으나, 탈락률 10%를 고려하여 131명에게 질문지를 배부하였다. 미회수된 질문지 6부와 불성실한 응답을 한 질문지 3부를 제외한 122부를 최종 분석하여 응답률은 93.1%이며, 최소 표본 수인 118명을 충족하였다. 본 연구의 선정 기준은 세계보건기구 기준 가임 여성의 연령인 49세 이하 여성, 인공 폐경, 조기 폐경, 자연 폐경이 안 된 여성, 연구 목적과 내용을 이해하고 연구에 자발적으로 참여하기를 동의한 여성이며, 제외 기준은 월경 주기와 관련된 내용을 조사하였기 때문에 임신 중 또는 수유 중인 여성은 제외하였다.

### 연구 도구

#### 월경 전 증후군

월경 전 약 2–10일에 시작하여 월경 시작 직전이나 월경 직후에 소실되는 것으로 일상생활에 지장을 줄 정도의 신체적, 정서적, 행동적으로 복합된 증후군을 의미하며, 본 연구에서는 총 19개 문항 Likert 4점 척도로 구성된 Heo [17]의 도구를 개발자로부터 사용 승인을 받은 후 사용하였다. 점수의 범위는 0점에서 57점으로 점수가 높을수록 월경 전 증후군의 증상이 심한 것을 의미한다. Heo [17]의 연구에서 신뢰도 Cronbach’s α=.86, 본 연구에서의 신뢰도는 Cronbach’s α=.92였다.

#### 내분비계 교란 물질 노출

내분비계 교란 물질 노출 위험 행위가 일상생활에서 얼마나 일어나는지를 의미하며, 본 연구에서는 Kim과 Kim [18]이 개발한 환경 호르몬 노출 저감화 행동 측정 도구에 “면 생리대를 사용한다”는 1항목을 추가하고, Kim과 Kim [18]의 항목 중 노출을 줄이는 저감화 행위는 역 환산하여 사용하였다. 본 연구에서 사용한 도구는 총 24문항 5점 척도로 구성되어 점수의 범위는 24점부터 120점으로 점수가 높을수록 내분비계 교란 물질 노출이 심한 것을 의미한다. 본 연구에서 사용한 수정 보완된 도구는 여성건강간호학 전공 교수 2인, 예방의학 교수 1인이 타당도를 검증하였으며, 면 생리대 사용에 대한 1문항이 추가된 24문항의 도구를 사용한 Park과 Park [16]에게 도구 사용 허락을 받아 사용하였다. Kim과 Kim [18]의 도구 개발 당시 신뢰도는 Cronbach’s α=.83, Park과 Park [16]의 연구에서는 Cronbach’s α=.86이었고, 본 연구에서의 신뢰도는 Cronbach’s α=.74였다.

#### 감정 노동

서비스를 거래하는 상황에서 조직이 요구하는 감정을 표현하는 데 필요한 노력, 계획, 통제를 의미하며, 본 연구에서는 Kim [19]의 도구를 도구개발자의 허락을 받아 사용하였으며, 총 9문항으로 구성된 Likert 5점 척도로 점수의 범위는 9점에서 45점으로 점수가 높을수록 감정 노동 강도가 높은 것을 의미한다. 도구의 신뢰도는 Kim [19]의 연구에서 Cronbach’s α=.86, 본 연구에서의 신뢰도는 Cronbach’s α=.87이었다.

#### 동료 지지

조직 내 직장 상사, 동료 및 후배로부터 받을 수 있는 정서적, 정보적, 물질적 지지로서, 본 연구에서는 Yang [20]의 도구를 이용하여 상사, 동료 및 후배 간호사로부터 받은 부서원의 사회적 지지를 측정하였다. 도구는 개발자의 사용 허락을 받은 후 사용하였으며, 총 20개 문항의 Likert 5점 척도이고 점수의 범위는 20점에서 100점으로 점수가 높을수록 동료 지지가 높음을 의미한다. Yang [20]의 도구 신뢰도는 Cronbach’s α=.94, 본 연구에서의 신뢰도는 Cronbach’s α=.95였다.

#### 일반적 특성 및 월경 관련 특성

간호사의 일반적 특성은 연령, 근무 부서, 결혼 여부를, 월경 관련 특성은 초경 나이, 월경 전 증후군 가족력, 월경량을 조사하였다. 월경량은 월경 혈액 손실을 평가하는 Higham 등[21]의 월경량 그림 평가도구를 사용하였다. 본 도구는 패드로 출혈량을 시각화하여 대상자가 하루에 사용하는 패드의 양을 기재하도록 되어있다. 패드의 경우 ‘조금’은 1점, ‘보통’은 5점, ‘매우 많음’은 20점으로 계산하고 총 점수가 많을수록 월경량이 많음을 의미하는데, 본 연구에서는 Higham 등[21]의 기준을 적용하여 점수가 100점 이상인 경우 월경 과다로 간주하였다.

### 자료 수집 절차

자료 수집 절차는 간호국에 연구 목적과 방법을 설명하고 자료 수집에 대한 승인을 얻은 후, 각 병동에 본 연구자가 직접 방문하여 병동 간호차장에게 연구 목적을 설명하고 대상자 선정 기준에 부합한 자에게 설문지 배부의 협조를 요청하였다. 설문지 회수는 연구자가 7–10일 후 각 병동을 방문하여 회수 봉투에 담겨 있는 설문지를 회수하였다. 자료 수집은 2019년 3월 22일부터 3월 31일까지 자가 보고형 설문지법을 이용하였으며, 응답 소요시간은 약 15–20분이었다.

### 자료 분석 방법

자료는 IBM SPSS Statistics for Windows ver. 23.0 (IBM Corp., Armonk, NY, USA) 프로그램을 이용하여 다음과 같이 분석하였다.

1) 대상자의 일반적 특성과 월경 관련 특성, 월경 전 증후군, 내분비계 교란 물질 노출 정도, 감정 노동, 동료 지지의 기술 통계로 실수와 백분율, 평균과 표준 편차를 산출하였다.

2) 일반적 특성에 따른 월경 전 증후군의 차이는 독립표본 t-test를 이용하였다.

3) 월경 전 증후군과 관련 요인들과의 상관관계는 연속형 변수는 Pearson’s correlation coefficient로, 이분형 변수는 Spearman’s correlation coefficient로 분석하였다.

4) 월경 전 증후군에 미치는 영향 요인은 위계적 다중회귀분석(hierarchical regression analysis)으로 분석하였다.

## Results

### 대상자의 일반적 특성 및 월경 관련 특성

대상자의 평균 연령은 28.90±6.92세였고, 20대가 73.8%로 대부분을 차지하였으며, 미혼이 98명(80.3%)이었다. 근무 부서는 일반 부서인 병동 근무 60명(49.2%), 외래 근무 14명(11.4%)이었으며, 특수 부서인 중환자실 근무 30명(24.6%), 응급실 근무 18명(14.8%)이었다. 초경 연령은 평균 12.95±1.59세였으며, 월경 전 증후군의 가족력이 있는 경우가 43명(35.2%)이었고, 월경량은 월경 과다가 78명(63.9%)이었다(Table 1).

### 내분비계 교란 물질 노출 정도, 감정 노동, 동료 지지 및 월경 전 증후군 정도

대상자의 내분비계 교란 물질 노출 정도, 감정 노동과 동료 지지 및 월경 전 증후군 정도는 Table 2와 같다. 내분비계 교란 물질 노출 정도는 총점 120점 만점에 평균 61.12±11.59점이었으며, 감정 노동은 총점 45점 만점에 평균 31.71±5.76점, 동료 지지는 총점 100점 만점에 평균 77.36±12.06점, 월경 전 증후군은 총점 57점 만점에 평균 17.01±9.07점이었다.

### 일반적 특성에 따른 월경 전 증후군 차이

대상자의 일반적 특성에 따른 월경 전 증후군 차이를 분석한 결과 중환자실과 응급실에 근무한 간호사들이 일반 병동과 외래에 근무하는 간호사보다(t=–2.59, *p*=.011), 월경 전 증후군 가족력이 있는 간호사가 가족력이 없는 간호사보다(t=2.01, *p*=.045), 월경량이 정상인 간호사보다 월경 과다인 간호사가(t=–3.04, *p*=.003) 통계적으로 유의하게 월경 전 증후군 정도가 높은 것으로 나타났다(Table 3).

### 월경 전 증후군과 관련 요인과의 상관관계

대상자의 월경 전 증후군과 주요 변인과의 관련성은 Table 4와 같다. 일반적 특성과 월경 전 증후군과의 상관관계에서는 중환자실과 응급실에 근무하는 경우(r_s_=.23, *p*=.011), 월경 전 증후군 가족력이 있는 경우(r_s_=.25, *p*=.007) 및 월경량(r=.20, *p*=.017)과 양의 상관관계가 있는 것으로 나타났다. 또한 월경 전 증후군은 내분비계 교란 물질 노출(r=.44, *p*<.001), 감정 노동(r=.45, *p*<.001)과 양의 상관관계를 보였고, 동료 지지(r=–.27, *p*=.003)와는 음의 상관관계를 보였다. 즉, 월경 전 증후군은 내분비계 교란 물질 노출 정도가 높을수록, 감정 노동이 높을수록, 동료 지지가 낮을수록 통계적으로 유의하게 높았다.

### 월경 전 증후군에 영향을 미치는 요인

월경 전 증후군에 영향을 미치는 관련 요인을 파악하기 위하여 위계적 다중 회귀분석으로 일반적 특성인 연령과 근무 부서를, 월경 관련 특성인 월경량과 월경 전 증후군 가족력을, 감정 노동과 동료 지지를, 내분비계 교란 물질 노출을 단계별로 투입하여 모형을 구성한 결과는 Table 5와 같다. 본 연구의 위계적 회귀분석 결과 공차 한계는 모두 0.1 이상의 수치를 보였고, 분산팽창지수(variance inflation factor)도 1.01–1.23으로 10을 넘지 않으므로 다중 공선성에 문제가 없는 것으로 판단하였다. Durbin-Watson은 2.15로 기준 값인 2에 가까운 수치를 나타내었으며, 0 또는 4와 가깝지 않아 독립 변수들 간의 상관관계가 없는 것으로 판단되어 회귀 모형은 적합하다고 할 수 있다.

제1 회귀 모형(F=3.98, *p*=.021)의 설명력은 5.0%였으며, 일반적 특성인 연령(t=–1.10, *p*=.271), 근무 부서(t=2.46, *p*=.015)로 근무 부서만 통계적으로 유의하였다. 제2 회귀 모형(F=5.20, *p*=.001)의 설명력은 12.0%로 9.0% 증가하였으며, 연령(t=–1.34, *p*=.182), 근무 부서(t=3.08, *p*=.003), 월경 전 증후군 가족력(t=2.27, *p*=.025), 월경량(t=2.38, *p*=.019)으로 통계적으로 유의한 변수는 근무 부서, 월경량, 월경 전 증후군 가족력 순이었다. 제3 회귀 모형(F=9.42, *p*<.001)의 설명력은 29.0%로 18.0% 증가하였으며, 연령(t=–1.47, *p*=.144), 근무 부서(t=3.14, *p*=.002), 월경 전 증후군 가족력(t=2.07, *p*=.040), 월경량(t=1.31, *p*=.192), 감정 노동(t=4.66, *p*<.001), 동료 지지(t=-1.94, *p*=.054)로 통계적으로 유의한 변수는 감정 노동, 근무 부서, 월경 전 증후군 가족력 순이었다. 제4 회귀 모형(F=11.74, *p*<.001)의 설명력은 38.0%로 9.0% 증가하였으며, 연령(t=–0.93, *p*=.350), 근무 부서(t=3.05, *p*=.003), 월경 전 증후군 가족력(t=2.65, *p*=.009), 월경량(t=1.36, *p*=.176), 감정 노동(t=3.48, *p*=.001), 동료 지지(t=–2.04, *p*=.043), 내분비계 교란 물질 노출(t=4.18, *p*<.001)로 통계적으로 유의한 변수는 내분비계 교란 물질 노출, 감정 노동, 근무 부서, 월경 전 증후군 가족력, 동료 지지 순이었다.

## Discussion

본 연구는 의료기관의 주요 인력인 간호사를 대상으로 월경 전 증후군의 관련 요인을 확인하였다. 월경 전 증후군은 증상이 심할 경우 결근과 생산성 저하를 가져와 직접적 혹은 간접적 의료비용을 증가시키는[22] 여성의 건강 문제이다. 그러므로 여성의 생식 건강의 지표가 되는 월경 관련 건강 문제는 여성 스스로가 알아서 해결해야 할 범주로 간주하는 것을 넘어서 직장이나 조직 내에서도 이를 완화시키기 위한 전략을 마련하는 것이 필요하다. 본 연구 결과 간호사의 월경 전 증후군의 관련 요인을 확인하기 위하여 투입된 위계적 회귀 모델을 설명하면 다음과 같다.

간호사의 월경 전 증후군에 영향을 주는 일반적 특성을 포함시킨 모델 1에서는 근무 부서가 응급실과 중환자실인 간호사들의 월경 전 증후군 정도가 심한 것으로 나타났다. 특히 응급실의 경우 보호자와 직접적인 대면이 많아 각종 불만에 노출되고 언어 폭력의 대상이 되기도 하여 자신의 감정을 억누르고 대상자를 대해야 하므로 감정 노동이 높은 부서로서[23], 감정 노동이 높을수록 스트레스가 높고, 스트레스가 높을수록 월경 전 증후군의 증상이 심하다는 선행 연구와 일치하였다[14]. 응급실이나 중환자실은 환자의 중등도가 높거나 위급한 상황 발생이 잦은 부서이므로, 이 곳에서 근무하는 간호사는 일반 병동 간호사보다 스트레스를 받을 수 있는 상황이 빈번하여 월경 전 증후군 증상을 더 심하게 호소하는 것으로 생각된다. 그러므로, 간호사의 부서 이동 시 개인의 부서 선호도와 특수 부서에서의 근무 기간 등을 확인하여 배치하는 전략이 필요할 것으로 생각된다.

또한 월경 전 증후군에 영향을 미치는 월경 관련 특성을 추가시킨 모델 2에서는 월경 전 증후군에 대한 가족력과 월경량이 관련요인으로 확인되었다. 월경 전 증후군의 가족력이 있는 경우 월경 전 증후군 증상이 더 심하다는 결과[16]와 일치하였는데 가족력은 유전적인 요인 뿐 아니라 고칼로리 섭취, 지방, 소금 및 설탕 섭취 등과 같은 식습관과 관련이 있다는 선행 연구를[9] 고려할 때, 월경 전 증후군의 개인적 중재뿐 아니라 가족 구성원 전체에게 상호 영향을 미칠 수 있는 식생활과 같은 생활 습관에 대해 가족 구성원을 포함하여 교육을 제공하는 전략도 필요함을 알 수 있다. 또한 월경량이 많은 경우 월경 전 증후군 증상이 더 심한 것으로 나타나 선행 연구 결과와도 일치하였다[4]. 월경량 과다는 다양한 여성 생식 건강 문제의 증상 중 하나이지만 월경량이 많은 간호사들은 월경 기간 동안 근무 시 생리 패드를 자주 교환하여야 하고, 활동량을 줄여야 하는 등 불편을 경험하게 된다[24]. 이러한 월경 과다로 인한 불편함은 월경 시작 2–10일 전에 나타나는 월경 전 증후군[2] 증상을 초래할 뿐만 아니라 월경에 대해 부정적인 태도[12], 부정적인 여성성을 가져온다[16]. 월경량 과다는 자궁 병변[24]으로 인해 나타날 수도 있는 증상이므로 이에 대한 경각심을 갖고, 병원을 방문해서 상세한 검사를 받도록 하는 등 여성 건강 관리가 필요하다고 본다.

모델 3에서는 간호사의 감정 노동은 월경 전 증후군에 통계적으로 유의하게 영향을 주는 것으로 확인되었다. 본 연구에서 감정 노동 점수는 45점 만점에 31.71점으로 나타났으며, 이 점수를 5점 척도로 환산하면 3.52점으로 Jun [25]의 연구에서의 3.42점보다 약간 높고 5점 척도의 중앙값인 2.50점은 상회하는 점수이다. 그러므로 의료계 환경이 고객 중심으로 변화하면서 최일선에서 대상자를 만나는 간호사들은 감정 노동이 심한 직업군이[11] 분명하다고 하겠다. 감정 노동은 심리적, 정서적인 영향을 넘어 신체적으로도 부정적 영향을 미치며[11], 생리 주기, 월경 전 증후군, 월경 통증, 임신, 산부인과적 증상과 관련된 생식 건강에도 영향을 미치는 것으로 보고되고 있다[26]. 감정 노동은 스트레스와 긴밀한 연관성이 있고, 스트레스 또한 월경 전 증후군과 밀접한 연관이 있는 것으로 알려져 있으며[12], Jun [25]의 연구에서도 간호사의 감정 노동이 완화되면 간호사의 신체적, 정신적, 사회적 건강 개선으로 직무 만족도 및 고객 만족도가 더욱 향상되어 간호의 질이 높아질 것이라고 제언하고 있어 간호사의 감정 노동을 줄이기 위한 다양한 전략은 반드시 필요하다고 하겠다.

내분비계 교란 물질 노출 정도를 포함시킨 최종 모델 4에서는 간호사의 월경 전 증후군 관련 요인이 내분비계 교란 물질 노출 정도, 감정 노동, 근무 부서, 가족력 및 동료 지지 순으로 나타나, 내분비계 교란 물질이 가장 영향력 있는 요인이었다. 선행 연구에 의하면 내분비계 교란 물질에 노출이 많을수록 체내에 축적된 물질들이 호르몬 생산에 영향을 미치므로 월경 전 증후군의 발생 빈도를 높인다고 보고하고 있다[6]. 내분비계 교란 물질 노출에 대한 위험 행위의 관련 요인으로 제시된 선행 연구를 살펴보면 내분비계 교란 물질 노출이 신체에 미치는 영향의 심각성에 대한 인식이 부족한 것으로 나타났으며[27], Seo와 Kim [28]의 연구에서도 내분비계 교란 물질의 위험성을 높게 인식할수록 내분비계 교란 물질에 노출되는 위험 행위가 감소하는 것으로 보고하고 있다. 따라서, 간호사들을 대상으로 내분비계 교란 물질 노출에 대한 위험성 인지 정도를 확인하고, 일상생활에서 내분비계 교란 물질 노출과 여성 건강이 관련된 근거를 제시하여 민감성을 높이는 정보 제공이 필요하다. 또한 월경 전 증후군 완화를 위해 내분비계 교란 물질에 노출을 줄일 수 있는 구체적인 행동을 안내해주는 프로그램을 개발하여 적용하는 것도 필요하다. 월경 전 증후군은 간호사의 건강에 영향을 미치고, 삶의 질과 근무 환경에서의 역할 수행에 영향을 미치며 나아가 환자들의 간호의 질에 영향을 줄 수 있으므로[2] 이를 완화할 수 있는 전략 개발은 매우 필요하다고 하겠다. 한편 동료 지지는 모델 3에서는 유의한 요인으로 제시되지 않았지만 모델 4에서는 유의한 변수로 제시되었다. 선행 연구에서도 동료 지지가 높을수록 직무 만족도가 높아 감정 노동이 감소되며[14], 동료 지지가 정서적인 안정을 주어 직무의 만족도를 높인다[29]고 보고하고 있다. 그러므로 동료 지지는 신체·정서·행동의 복합적 증후군인 월경 전 증후군과 같은 간호사의 건강 문제를 완화할 수 있는 전략으로 활용되어야 함을 알 수 있다.

본 연구는 근무 관련 특성인 감정 노동과 근무 부서가, 그리고 생활습관인 내분비계 교란 물질 노출 정도가 간호사의 월경 전 증후군에 영향을 미치는 것으로 확인하였다는 데 의의가 있다. 이러한 결과는 교육적 측면에서는 내분비계 교란 물질이 포함된 물품에 대한 정확한 정보를 제공하여 일상생활에서 노출을 최소화할 수 있도록 보건 교육에 활용할 수 있으며, 실무적 측면에서는 간호사의 감정 노동을 줄이기 위해 병동 책임자가 간호사의 개인 역량을 고려한 업무 배정 및 부서 내 동료 지지 프로그램을 개발 적용하여 간호사의 월경 전 증후군 완화에 도움을 줄 뿐 아니라 직무 만족도를 높이는 데 활용할 수 있다고 본다. 본 연구는 한 대학병원에 근무하는 간호사를 대상으로 월경 전 증후군의 관련 요인을 확인한 점에서 일반화에는 제한이 있지만, 월경 전 증후군과 가장 관련성이 높은 변수가 내분비계 교란 물질임을 확인하였다. 내분비계 교란 물질 노출에 대한 인식은 생활 속에서 유해 화학 물질을 피하며 채식 위주의 식사와 유기농 식품을 선택하는 등의 행동으로 내분비계 교란 물질에의 노출로 인한 건강 문제의 발생을 줄일 수 있는 것으로 제시되었다[30]. 이에 친환경 생활양식과 월경 전 증후군과의 관련성을 확인하는 연구를 제언한다. 또한 본 연구에서 사용한 내분비계 교란 물질 노출 측정 도구는 일상생활에서 노출되는 내용을 측정하는 도구이므로, 보건 의료 환경을 반영하여 측정할 수 있는 내분비계 교란 물질 노출 정도를 측정하는 도구 개발 연구를 제언하는 바이다.

## Figures and Tables

**Table 1.** Distribution of participants’ characteristics (N=122)

Characteristic	Categories	n (%)	Mean±SD
Age (year)	≤29	90 (73.8)	28.90±6.92
	≥30	32 (26.2)	
Working department	General unit		
	Ward	60 (49.2)	
	OPD	14 (11.5)	
	Special unit		
	ICU	30 (24.6)	
	ER	18 (14.8)	
Marital status	Unmarried	98 (80.3)	
	Married	24 (19.7)	
Age at menarche (year)			12.95±1.59
Family history of premenstrual syndrome	Yes	43 (35.2)	
	No	79 (64.8)	
Amount of menstruation	Normal	44 (36.1)	
	Menorrhagia	78 (63.9)	

ER: Emergency room; ICU: intensive care unit; OPD: outpatient department.

**Table 2.** Exposure to endocrine disruptors, burnout, social support from peers, and premenstrual syndrome among participants (N=122)

Variable	Mean±SD	Number of items	Item range	Possible range
Exposure to endocrine-disrupting chemicals	61.12±11.59	24	1–5	24–120
Burnout	31.71± 5.76	9	1–5	9–45
Social support from peers	77.36±12.06	20	1–5	20–100
Premenstrual syndrome	17.01± 9.07	19	0–3	0–57

**Table 3.** Comparison of premenstrual syndrome according to participants’ characteristics (N=122)

Characteristic	Categories	Mean±SD	t (*p*)
Age (year)	≤29	16.43±8.83	–0.41 (.676)
	≥30	17.22±9.19	
Working department	General unit^†^	15.33±8.13	–2.59 (.011)
	Special unit^‡^	19.60±9.88	
Marital status	Unmarried	17.29±9.46	0.66 (.510)
	Married	15.91±7.35	
Family history of premenstrual syndrome	Yes	19.12±9.34	2.01 (.045)
	No	14.34±8.76	
Amount of menstruation	Normal	13.79±8.21	–3.04 (.003)
	Menorrhagia	18.83±9.07	

† General unit: outpatient departments and wards.

‡ Special unit: emergency room and intensive care unit.

**Table 4.** Correlations between premenstrual syndrome and related factors (N=122)

Variable	r/r_s_(*p*)						
	Age	Working department	Family history of PMS	Amount of menstruation	Exposure of EDCs	Burnout	Social support from peers
Working department^†^	–.03 (.721)						
Family history of PMS^†^	.02 (.824)	–.17 (.061)					
Amount of menstruation	07 (.451)	.04 (.680)	.17 (.062)				
Exposure to EDCs	–.21 (.014)	.16 (.076)	–.19 (.032)	.21 (.014)			
Burnout	–.20 (.017)	.23 (.011)	.03 (.767)	.24 (.009)	.35 (<.001)		
Social support from peers	.04 (.681)	–.17 (.061)	.01 (.890)	–.06 (.468)	–.05 (.547)	–.48 (.<.001)	
PMS	–.13 (.177)	.23 (.011)	.25 (.007)	.20 (.017)	.44 (<.001)	.45 (<.001)	–.27 (.003)

EDCs: Endocrine-disrupting chemicals; PMS: premenstrual syndrome.

† Spearman correlation coefficient.

**Table 5.** Factors influencing premenstrual syndrome (N=122)

Variable	Model 1			Model 2			Model 3			Model 4		
	B	β	t (*p*)	B	β	t (*p*)	B	β	t (*p*)	B	β	t (*p*)
Constant	19.15		5.31 (<.001)	15.42		4.25 (<.001)	7.13		0.98 (.329)	–5.02		–0.67 (.499)
Age	–0.12	–0.09	–1.10 (.271)	–0.15	–.11	–1.34 (.182)	–.148	–0.11	–1.47 (.144)	–0.09	–0.06	–0.93 (.350)
Working department^†^	4.06	0.22	2.46 (.015)	4.96	0.26	3.08 (.003)	4.57	0.24	3.14 (.002)	4.16	0.22	3.05 (.003)
Family history of premenstrual syndrome^†^				3.736	0.19	2.27 (.025)	3.07	0.16	2.07 (.040)	3.7	0.19	2.65 (.009)
Amount of menstruation				0.02	0.2	2.38 (.019)	0.01	0.1	1.31 (.192)	0.01	0.1	1.36 (.176)
Emotional labor							0.59	0.37	4.66 (.000)	0.43	0.27	3.48 (.001)
Support from peers							–0.11	–0.15	–1.94 (.054)	–0.11	–0.15	–2.04 (.043)
Exposure to endocrine-disrupting chemicals										0.25	0.32	4.18 (<.001)
F(df), *p*	3.98(1), 021			5.20(3), 001			9.42 (5), <.001			11.74 (6), <.001		
Adjusted R²	.05			.12			.29			.38		
R² change				.09			.18			.09		

† The dummy variable references were working department (emergency room and intensive care unit) and family history of premenstrual syndrome (yes).
