# Addendum: Effects of exposure to endocrine disruptors, burnout, and social support from peers on premenstrual syndrome in nurses

**DOI:** 10.4069/kjwhn.2020.09.22

**Published:** 2020-09-22

**Authors:** Hye Young Chang, SoMi Park

**Affiliations:** 1 Division of Nursing, Wonju Severance Christian Hospital, Wonju, Korea; 2 Department of Nursing, Yonsei University Wonju College of Medicine, Wonju, Korea

This addendum was issued to note the statistical information for the R2 change values in Table 5 of the following article:

Chang HY, Park SM. Effects of exposure to endocrine disruptors, burnout, and social support from peers on premenstrual syndrome in nurses. Korean J Women Health Nurs. 2020;26(2):171-179. https://doi.org/10.4069/kjwhn.2020.06.18

## Figures and Tables

**Table:** Factors influencing premenstrual syndrome (N=122)

Variable	Model 1			Model 2			Model 3			Model 4		
	B	β	t (*p*)	B	β	t (*p*)	B	β	t (*p*)	B	β	t (*p*)
Constant	19.15		5.31 (<.001)	15.42		4.25 (<.001)	7.13		0.98 (.329)	–5.02		–0.67 (.499)
Age	–0.12	–0.09	–1.10 (.271)	–0.15	–.11	–1.34 (.182)	–.148	–0.11	–1.47 (.144)	–0.09	–0.06	–0.93 (.350)
Working department^†^	4.06	0.22	2.46 (.015)	4.96	0.26	3.08 (.003)	4.57	0.24	3.14 (.002)	4.16	0.22	3.05 (.003)
Family history of premenstrual syndrome^†^				3.736	0.19	2.27 (.025)	3.07	0.16	2.07 (.040)	3.7	0.19	2.65 (.009)
Amount of menstruation				0.02	0.2	2.38 (.019)	0.01	0.1	1.31 (.192)	0.01	0.1	1.36 (.176)
Emotional labor							0.59	0.37	4.66 (.000)	0.43	0.27	3.48 (.001)
Support from peers							–0.11	–0.15	–1.94 (.054)	–0.11	–0.15	–2.04 (.043)
Exposure to endocrine-disrupting chemicals										0.25	0.32	4.18 (<.001)
F (df), *p*	3.98(1), 021			5.20(3), 001			9.42 (5), <.001			11.74 (6), <.001		
Adjusted R²	.05			.12			.29			.38		
R² change (F, p)				.09 (6.08, .003)			.18 (15.32,<.001)			.09 (6.40,.002)		

† The dummy variable references were working department (emergency room and intensive care unit) and family history of premenstrual syndrome (yes).

